# Barriers and facilitators in the provision of post-abortion care at district level in central Uganda – a qualitative study focusing on task sharing between physicians and midwives

**DOI:** 10.1186/1472-6963-14-28

**Published:** 2014-01-21

**Authors:** Mandira Paul, Kristina Gemzell-Danielsson, Charles Kiggundu, Rebecka Namugenyi, Marie Klingberg-Allvin

**Affiliations:** 1 Department of Women’s and Children’s Health, Karolinska Institutet, University Hospital, Stockholm, Sweden; 2 Department of Women’s and Children’s Health, IMCH, Uppsala University, Uppsala, Sweden; 3 Department of Obstetrics and Gynaecology, Mulago Hospital, Kampala, Uganda; 4 Department of Public Health Sciences, Makerere University, Kampala, Uganda; 5 School of Social and Health Science, Dalarna University, Falun, Sweden

**Keywords:** Induced abortion, Post-abortion care, Task sharing, Uganda, Contraceptive counselling, Misoprostol

## Abstract

**Background:**

Abortion is restricted in Uganda, and poor access to contraceptive methods result in unwanted pregnancies. This leaves women no other choice than unsafe abortion, thus placing a great burden on the Ugandan health system and making unsafe abortion one of the major contributors to maternal mortality and morbidity in Uganda. The existing sexual and reproductive health policy in Uganda supports the sharing of tasks in post-abortion care. This task sharing is taking place as a pragmatic response to the increased workload. This study aims to explore physicians’ and midwives’ perception of post-abortion care with regard to professional competences, methods, contraceptive counselling and task shifting/sharing in post-abortion care.

**Methods:**

In-depth interviews (n = 27) with health care providers of post-abortion care were conducted in seven health facilities in the Central Region of Uganda. The data were organized using thematic analysis with an inductive approach.

**Results:**

Post-abortion care was perceived as necessary, albeit controversial and sometimes difficult to provide. Together with poor conditions post-abortion care provoked frustration especially among midwives. Task sharing was generally taking place and midwives were identified as the main providers, although they would rarely have the proper training in post-abortion care. Additionally, midwives were sometimes forced to provide services outside their defined task area, due to the absence of doctors. Different uterine evacuation skills were recognized although few providers knew of misoprostol as a method for post-abortion care. An overall need for further training in post-abortion care was identified.

**Conclusions:**

Task sharing is taking place, but providers lack the relevant skills for the provision of quality care. For post-abortion care to improve, task sharing needs to be scaled up and in-service training for both doctors and midwives needs to be provided. Post-abortion care should further be included in the educational curricula of nurses and midwives. Scaled-up task sharing in post-abortion care, along with misoprostol use for uterine evacuation would provide a systematic approach to improving the quality of care and accessibility of services, with the aim of reducing abortion-related mortality and morbidity in Uganda.

## Background

Unsafe abortions are estimated to account for 13% of maternal deaths globally. They give rise to a large number of short- and long-term complications [1]. An estimated 21.9 million unsafe abortions are performed in the world annually [2], and 97% of these occur in low-income countries. Africa is responsible for the second largest proportion of unsafe abortions (44%), and the highest rates globally (18–39 per 1,000 women) [3]. Abortion laws of many countries are restrictive, leaving women no choice other than to procure unsafe abortion [1]. General access to and use of contraceptive methods are limited, and the low status of women prevents them from making independent decisions on their own sexual and reproductive health (SRH) [4].

Abortion is defined as the termination of a pregnancy, whether spontaneous, occurring before 22 weeks of gestation [5], or induced [6]. The World Health Organization (WHO) defines unsafe abortion as any procedure with the purpose of terminating a pregnancy that is performed by persons lacking the proper skills and/or that is performed in an unhygienic, non-medical setting [2]. To address the complications related to incomplete, spontaneous or unsafely-induced abortions, post-abortion care (PAC) has been introduced in countries where abortion laws are restrictive. Standard PAC includes emergency care (such as resuscitation using blood transfusions, intravenous lines, antibiotics etc.); contraceptive counselling; treatment of sexually transmitted infections; human immunodeficiency virus (HIV) counselling; and community empowerment [7].

The recommended method for treatment of an incomplete abortion during the first trimester is manual vacuum aspiration (MVA) [5] or prostaglandin E1 analogue *misoprostol*[8-10]. International studies have compared the two methods (misoprostol and MVA) for the treatment of incomplete abortions, and the results show no significant difference in their effectiveness [11-15]. Recent studies indicate the safety and efficiency of misoprostol use instead of surgical interventions to treat incomplete abortions in low-resource settings [10,16,17]. Misoprostol has therefore been suggested as a first-line therapy in the developing world [18], although there are still barriers to the accessibility of the drug [19,20].

Shortage of human resources in the health care system is common in low-income countries, especially in remote areas where maternal mortality is high [21]. The strategic use of mid-level providers in the sense of task shifting/sharing – a process of delegating tasks to less specialized health care providers – has been identified as something that increases productivity and efficiency within health systems [22,23]. A task shift in the provision of treatment for incomplete abortion will increase women’s access to PAC [24]. However, along with structural arrangements it is important to consider the providers’ attitudes to the tasks they are to provide. Attitude, beliefs and experience of health care providers have shown to influence the provision of post-abortion care [25-28]. Moreover, training and experience has a positive effect on attitudes and it facilitates the bridging of cultural beliefs with the reality of service provision [29,30].

### The Ugandan context

Uganda has one of the highest total fertility rates (6.7) in the world [31], and it is estimated that 56% of all pregnancies are unintended. The contraceptive prevalence is 23% [32] and the unmet need for contraceptives is 33% among women of reproductive age [4]. Several reasons for the poor uptake of contraception are reported, for example misconceptions, side effects and poor acceptance [33,34]. Induced abortion is restricted and permitted by law only to save the life of a woman. Still, an estimated 297,000 induced abortions are performed annually, resulting in an overall abortion rate of 54 per 1,000 women aged 15–49 [35]. This is regarded as high compared to the estimated rate for Eastern Africa [35,36]. About 77% of abortions treated in the public health system are induced [35]. Additionally, unsafe abortions account for almost 40% of admissions to emergency obstetric care units, and they are responsible for significant morbidity and mortality among women [37].

The National Policy Guidelines and Service Standards for Sexual and Reproductive Health and Rights of 2006 [38] indicates that PAC is implemented in Uganda for treatment of incomplete abortion caused by spontaneous or induced abortion. In addition, the policy enables a variety of providers (midwife/nurse, clinical officer, medical officer and gynaecologist) to treat incomplete abortions. In spite of this progressive policy, mid-level providers such as nurses and midwives largely lack the proper training to provide PAC. This shortage of qualified providers limits the availability of safe emergency obstetric care, including PAC [22,39]. The 2006 SRH policy also permits abortions when the woman’s life is endangered, and in cases of foetal anomaly, rape, incest, cervical cancer, or upon a request from an HIV positive woman [38]. However, the Ugandan Constitution states that abortion is only allowed if the procedure is authorized by law. Consequently, interpretations of the law are ambiguous, and providers may be reluctant to perform an abortion for fear of the legal consequences [39]. A study from 2005 suggests that the most common method used to treat post-abortion complications in governmental as well as private facilities in Uganda was curettage for uterine evacuation [4], a method that is dismissed by the WHO [5]. It is suggested that optimizing the use of primary health care providers other than physicians, by task shifting, would be cost-effective and decrease the unnecessarily high maternal mortality in Uganda [22]. However, the utilization of task sharing and shifting is poorly monitored, and little is known of the extent to which mid-level providers are involved. The acceptability and perception of PAC among providers at district level in Uganda has not been adequately explored. Moreover, providers’ needs of training and the challenges they face in the provision of PAC need to be better understood. This may help to overcome the barriers on provider-level, to better implement task sharing and finally to improve quality PAC in Uganda. Attending to the issue of unsafe abortion and PAC is a crucial step towards decreasing the maternal mortality ratio (MMR) in Uganda.

### Aim of study

To explore physicians’ and midwives’ perceptions of post-abortion care, with regard to professional competences, methods, contraceptive counselling and task shifting/sharing in PAC.

## Methods

### Study design

An inductive study approach using an emergent design was employed, utilizing the qualitative method of in-depth interviews (IDI). Thematic analysis was used to structure the data.

### Study setting

The study was performed at seven health care facilities situated in five different districts in the Central Region of Uganda. The region was chosen because the abortion rate has been reported to be the second highest in the country, and it is above the national average (62 per 1000 women) [36]. The seven facilities were purposely selected because of their high caseloads in PAC. The caseloads were mapped through a survey done by one of the Ugandan researchers prior to the initiation of this study (2012). In addition, the employment of both doctors and midwives in a facility was regarded a criterion for inclusion, and we aimed to include facilities from rural, semi-urban and urban settings. The Ugandan health system is made up of a national referral hospital, regional and district hospitals, and health centres II-IV. Health centre IV is the most advanced, employing both doctors and midwives, and health centre II is the least advanced, employing a nurse or a clinical officer. Health centre I, also referred to as village health teams, are the lowest level and have no permanent accommodation [40].

### Study participants

A total of 27 IDI were carried out with health care providers in the health facilities listed above (Table 1). The majority of respondents (all the midwives and one doctor) were female (70%). The midwives had all worked for eight or more years, while the work experience of the doctors ranged between one and twenty years. The health facilities selected were staffed by doctors and midwives, and were equipped to provide basic and comprehensive emergency obstetric care in rural, semi-urban and urban areas. National, regional, district and sub-district levels were included (Table 1) to explore any discrepancies in service provision among the levels as well as differences between the cadres.

**Table 1:** Facilities included in data collection

**Facility**	**Setting/Area**	**Level**	**Doctors interviewed**	**Midwives interviewed**
Mpigi	Rural	Health Centre IV	1	1
Luweero	Rural	Health Centre IV	1	3
Nakaseke	Semi-urban	District Hospital	1	4
Gombe	Semi-urban	District Hospital	1	2
Entebbe	Urban	District Hospital	2	2
Masaka	Urban	Regional Hospital	2	3
Mulago	Urban	National Hospital	2	2 (pilot)
**Total**			**10**	**17**

The table shows the location, setting and level of health facility as well as the number of study subjects participating in the IDI from each health facility, including the distribution between doctors and midwives.

#### Inclusion criteria

1. Being employed in one of the hospitals/Health centres listed above (Table 1)

2. Being a nurse, a midwife, a clinical officer or a doctor

3. Actively participating in PAC

### Data collection

The IDI were conducted in February and March 2012, and continued until no new data were encountered. Purposive sampling was employed. The person in charge at each health facility, such as the medical officer or head nurse/midwife, was used as a gatekeeper, facilitating the identification of eligible study subjects. All participants signed a written consent prior to the IDI, and the interviews were conducted in the hospital, meaning that interruptions could sometimes occur. Two pilot interviews were performed at Mulago hospital to test the interview guidelines, resulting in a revision to impart a better in-depth character to the questions. The final guideline was semi-structured, open-ended and utilized probes. Topics covered included (i) attitudes towards abortion, PAC and family planning, (ii) perceptions of methods for uterine evacuation, (iii) skills and competences needed and (iv) task sharing in PAC. The IDI were performed in English, lasted for 30–60 minutes and were tape-recorded. The research team consisted of researchers with different cultural and professional backgrounds. The first author and main researcher, a Swedish woman with a background in global health, and a Ugandan assistant, a woman with a degree in public health, conducted the data collection. All the researchers were involved in the analysis and interpretation of the data.

### Data analysis

The recorded data were transcribed verbatim, read through several times and carefully coded manually. Since little is known about the views and perceptions on PAC in Uganda, thematic analysis was used [41]. Furthermore, a thick description to reflect the content of the data was employed, and codes were organized into sub-themes using an inductive approach. Sub-themes were arranged into semantic themes focusing on identified patterns as well as on the broader meanings and implications of the data. The analysis was done with a realistic or epistemological approach, looking at experience and meaning in a straightforward way. The interpretation of the data was continuously discussed and re-evaluated by all researchers in the team. Additionally, the data were reviewed to identify extracts for illumination of the themes.

### Ethical considerations and confidentiality

Ethical approval was obtained from the Makerere University College of Health Sciences, School of Biomedical Sciences Research and Ethics Committee, Kampala. National approval was given by the Uganda National Council for Science and Technology. The study was identified as a minimal risk study since it addressed health care providers’ perspective on a work task, and their opinions on abortion. A written consent form stated the rights of the participants, and confidentiality and the anonymity of the participants were guaranteed. The data and informed consent forms are stored safely under lock and key at Karolinska Institutet and are used only by the researchers involved in the study.

## Results

**Figure 1:**
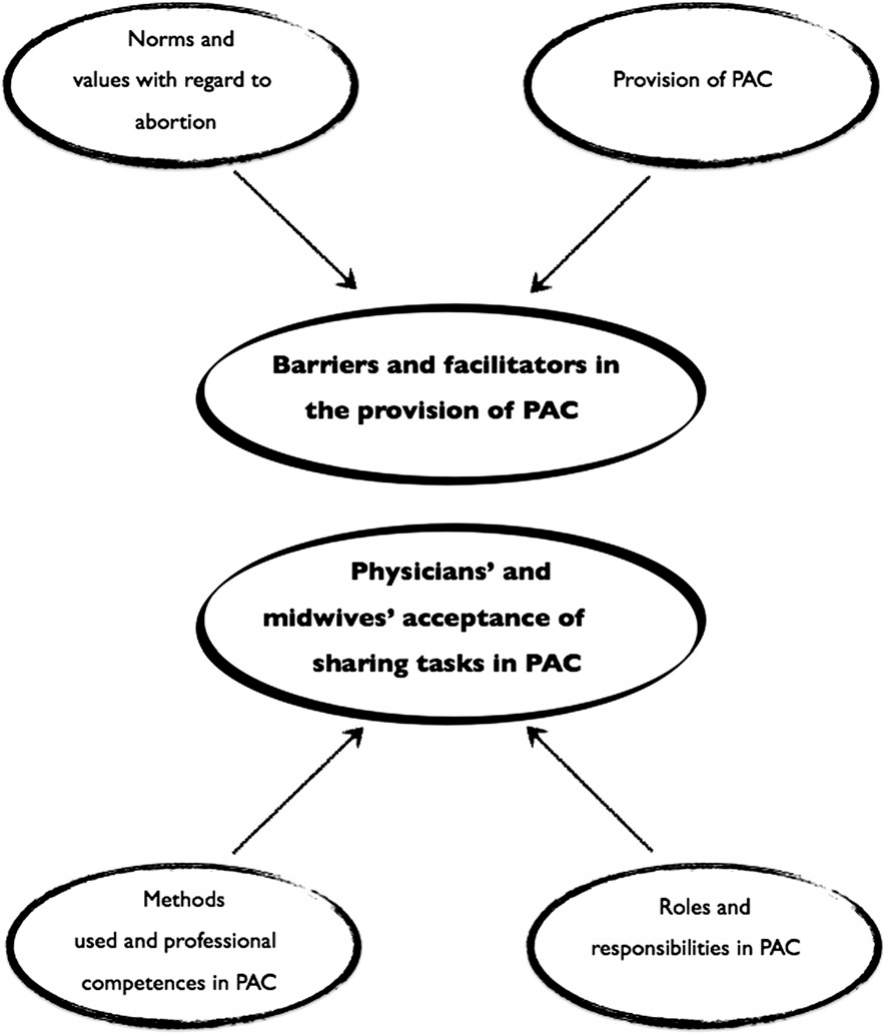
**Thematic map.** Thematic map, illustrating the sub-themes and the two final overarching themes of barriers and facilitators in the provision of PAC, and physicians’ and midwives’ acceptance of sharing tasks in PAC.

The analysis resulted in two main themes that emerged from the four sub-themes illustrated in Figure 1 and presented in the text below.

### Barriers and facilitators in the provision of post-abortion care

#### Norms and values with regard to abortion

The informants described different types of abortion: spontaneous abortion, induced abortion, criminal abortion and safe abortion. Spontaneous abortions were described as uncontrollable and undesired, and perceived to need extra care. Induced abortions were sometimes perceived as unsafe, when carried out under clandestine circumstances, and sometimes as safe, when carried out in a hospital by skilled personnel. Criminal abortion was always referred to as clandestine and unsafe, and as resulting in complications. The perceptions about safe abortions were vague, and scepticism about whether they really exist was prominent. However, opinions that MVA was the only safe method, or that safe abortion could be performed up to 12 weeks, were also expressed.

*But what is safe abortion? Maybe I do not have the knowledge about safe abortion when it is being induced. I don’t know how safe it is. Maybe I need to know more about it.* (Midwife, 12 years’ experience)

*The abortions they go to procure are very unsafe and they end up with them here, disturbing you. Now, because in our society it is done in secrecy (…) And these ones end up with problems of course (…) once she has decided she doesn’t want this pregnancy, whatever you do, she’ll do it.* (Doctor (ob./gyn.), 14 years’ experience)

When questioned about their opinions and their feelings about induced abortion, the respondents rarely approved of the practice, and abortion was commonly perceived as immoral, and sometimes as murder. However, informants felt sad to see young women suffer from severe complications caused by unsafely-induced abortions. All agreed that, when carried out with proper motives, abortion could be acceptable, e.g. when the life of the woman was in danger and in cases of rape. A more liberal view, given by the experienced doctors and specially trained midwives, was that there could be a number of reasons leading to a woman procuring an abortion.

*…You have lost your uterus, your health will not remain the same and you will never have a good future because you will not get married and get a child, hmm. So it is really sad, it is really sad and it is very common.* (Senior medical officer, 12 years’ experience)

*God forbid, I wouldn’t (…) I’ve never and I would not! (…) Some hard-hearted fellows can do that, not me. This is murder, you’re macerating, distorting, mutilating this unborn child, it’s degrading of human beings.* (Doctor/intern, 1 year’s experience)

When addressing the question of whether or not abortion should be legalized, different opinions were communicated. Increased workload, long waiting times and the perception that Uganda is not yet ready for such a change came forward as opposing arguments. Those in favour of legalization argued that MMR would decrease as a consequence, but that the social stigma would continue to limit the number of induced abortions, so that legalization would not result in an increased workload, but rather in better-controlled and safer health care. Doctors who admitted to having carried out abortions preferred not to do so again since they ended up feeling guilty, and got the blame when something went wrong. On the other hand, doctors and midwives expressed satisfaction in helping women to abort when the reason was legitimate and they felt safe providing the abortion. This was commonly justified by the experience that the woman would seek help elsewhere if neglected by the health facilities, resulting in an unsafely-induced abortion and complications. Midwives would perceive abortion as a work task if it were legalized, and would not feel compromised conducting it, but a reluctance to bear the guilt of killing and a fear of being judged by God as well as by society were present amongst some of the providers.

*Uganda, if they legalized abortions, it would be terrible. It will encourage many girls to be engaged in sexual activity because if they get pregnant, women will abort and no one will say no, you know.* (Head nurse/midwife, 15 years’ experience)

*…Faith is strong among the Africans, so it would be a strong resistance to pass through, but, if it is legalized, I believe it would save lives, and then it would save people from the burden of taking care of a child that is unplanned and it would (…) help people solve that burden.* (Doctor, 10 years’ experience)

*After knowing that there are safe abortions, and I’m doing it and I’ve done it, so I would advice what? It’s her choice if she doesn’t want it. Even if I refuse her to do it, she will end up doing criminal…* (Midwife, 10 years’ experience)

All agreed that if induced abortion were to be legalized, there would be an increased need for human resources as well as a special unit and available instruments to provide safe abortions. Moreover a wish to choose whether or not to provide the service was expressed.

#### Provision of post-abortion care

Post-abortion care was described as emergency care and contraceptive counselling. Some of the midwives had difficulties defining it, but, when asked about the procedures and methods, they were generally able to explain these. The common attitude towards PAC was positive, and informants saw it as necessary. No issue of blame or sin from the providers’ side among the midwives or doctors was brought up in the context of PAC, since the health care providers themselves were not involved in conducting the abortion. However, midwives in particular expressed frustration with patients who had had unsafely-induced abortions. This was explained by the additional workload resulting from the self-induced procedures. Another commonly acknowledged source of frustration was when a woman denied having procured an abortion and told lies about what had happened, increasing the assessment time and complicating the service provision. This, in combination with the workload, could lead to harsh treatment by the midwives. However, there was also an expression of sadness among the providers when describing the severity of the complications experienced by young girls.

*We see so many come when they have inserted sticks, they’ve inserted herbs, they have inserted miso (misoprostol). This is also adding to my workload. She knew exactly what she was doing and she is adding, so the attitude (among providers) is not so good, especially not with criminal (abortion).* (Head nurse/midwife, 15 years’ experience)

Health care providers’ bad attitude when caring for women in need of PAC was discussed. Patronizing and judgemental behaviour was said to be more common towards women with suspected induced abortion, and this affected the quality of care. All informants stressed that women were equally cared for, that there was no discrimination and that money was not a deciding factor. However, when the choice was given, a woman with spontaneous abortion would be given priority to a woman with induced abortion, if their complications were equally severe. Experience and in-service training were cited as ways of promoting attitude change, and so more education and training were suggested and desired.

Family planning in relation to PAC was perceived as important to avoid another induced abortion or unintended pregnancy. However, PAC and family planning were rarely provided in the same clinical unit, and patients would be referred to family planning after counselling and discharge from the PAC unit. Midwives complained about not having enough time or space to provide proper PAC counselling, including contraceptive counselling. Neither did they have the resources to follow up discharged PAC patients to see whether family planning advice was properly provided. Moreover, doubts about what methods would be appropriate after abortion were prevalent, especially for young unmarried girls.

*…Now the patients we see are not counselled, (…), we are completing the abortion for you and then you’ll leave, go! Go to family planning. And there’s no nurse for her there. The nurse who is doing ANC is the one who will do the family planning, you find her counselling mothers, she doesn’t have time to sit with this young girl who has had an abortion. So they are not being attended to effectively, hmm.* (Doctor (ob./gyn.), 14 years’ experience)

Only one informant claimed to provide family planning along with PAC, however with no great success.

*We normally provide (family planning) before discharge apart from a few who escape. By the way, those post-abortals, they escape; normally they do not wait for discharge.* (Midwife, 20 years’ experience)

### Physicians’ and midwives’ acceptance of sharing tasks in post-abortion care

#### Methods used and professional competences in PAC

Manual vacuum aspiration was stated to be the first choice of method for uterine evacuation in first-trimester abortions, while dilatation and curettage (D&C) would be used in the second trimester. Misoprostol was rarely mentioned (except when the interviewer specifically asked about it) and was seldom used at district level for PAC.

*Depending on gestational age, below 12 weeks MVA is the best, above 12 weeks we can use curettage (D&C)… miso is not common in this country, it’s not available, it’s still expensive…* (Doctor, 14 years’ experience)

*It depends on the gestation age. If it’s first trimester I’d prefer MVA and if second I would prefer using miso.* (Doctor/intern, 1 year’s experience)

MVA could not be performed in all health facilities because of a lack of equipment and trained midwives. Moreover, in facilities where trained staff was available, there were not always sufficient MVA kits. This resulted in an overuse of the existing equipment and questionable safety of the procedure.

*… It’s curettage, we do curettage, we do all that, that’s the major one we have here, we don’t have this other thing of … (interviewer: MVA?) aha, we don’t have that one.* (Senior medical officer, 12 years’ experience)

Midwives trained in MVA found it efficient, with satisfactory outcomes. Women could be discharged the same day and incomplete abortions were successfully completed. One doctor was unconvinced that MVA was efficient and preferred D&C, whereas other doctors supported the use of MVA, however they would still prefer D&C if they, themselves were to be in charge of the uterine evacuation. Misoprostol was known for the treatment of postpartum haemorrhage, intra-uterine foetal death and induction of labour, but was sometimes also used in PAC. Providers trained in misoprostol use for PAC were positive about the method and preferred it to MVA.

*There’s no risk of perforation, no risk of infection, it (misoprostol) has very many, very few risks compared to introducing foreign bodies in the uterus. I prefer, and it’s very effective, so effective.* (Doctor/intern, 1 year’s experience)

Furthermore, these respondents aware of misoprostol use found it suitable for provision at any level of the health system and by any health care provider, when trained. Midwives were keen to learn more about it, while doctors were more interested in further training in PAC counselling including family planning. One doctor considered in-service training a waste of time and money when not exclusively for doctors.

The poor use of misoprostol in PAC was due to a lack of knowledge among providers, no hospital guidelines and a lack of available drugs in the health facilities. Patients have to buy the drug from private providers and misoprostol was perceived as expensive and difficult to access.

Additionally, insecurity among midwives in terms of side effects was common, and a discomfort about discharging a woman who is in pain or bleeding was communicated. There was a prominent perception that misoprostol induces a slow and painful reaction, with a great blood loss and continuous bleeding for weeks. Furthermore, women seeking PAC were commonly eager to get immediate treatment, and the respondents thought that misoprostol would not be applicable. Additionally, the lack of a guarantee of a completed abortion unless an ultrasound was done contributed to midwives’ preference for MVA for first-trimester abortions, so that they were in control of the procedure. Moreover, a fear of misuse of misoprostol, if it was accessible, was communicated.

*…it (misoprostol) takes long for the bleeding to stop, somebody goes around trickling for about a week, what if I would do an MVA? Actually, when you do an MVA there should be no more bleeding within the next few hours. That’s what I don’t like with miso (…) And the other thing (…) people are misusing it (…) they give it to pregnant women and they end up with ruptured uterus, we actually lost a mother last week because of that…* (Doctor (ob./gyn.), 20 years’ experience)

*We limit its (misoprostol’s) use, because if people who work with us get to know that we are using this thing (misoprostol) and it does these wonders, we would get a problem. (*Senior medical officer, 12 years’ experience)

#### Roles and responsibilities in PAC

All informants agreed that midwives were the key actors in PAC. They received and assessed the patient, called for a doctor if necessary and gave counselling upon discharge. Doctors were commonly absent, in which case midwives preferred to treat the patients alone to prevent their conditions from worsening.

Midwives with some experience of MVA would provide it, with or without official training, and in the more complicated cases a doctor would be called for. In the absence of a doctor, two of the midwives would perform D&C, since they had been trained by a doctor, and one midwife explained that she would do manual removal of the placenta followed by MVA. Midwives with no training or knowledge would not provide MVA, but would refer the patient to a doctor for D&C or send her to another hospital. However, this was perceived to be frustrating since the life of the woman would be endangered and the patient would not always have the means of transport for the referral. Misoprostol was prescribed and administered by doctors when available. Midwives sometimes treated patients with misoprostol, although commonly after consulting a doctor.

*Curettage is only me (…) but majority of midwives do MVA (…) The doctors? They can do it if they are around. But you know this mother who comes bleeding and the doctor is not around, so you want to save her, and if you know the procedure, why do you wait for the doctor? So you carry out (the procedure); you provide the mother with antibiotics, then you can inform the doctor…* (Midwife, 12 years’ experience)

*Doctors are few (…) so most midwives have been trained how to do MVA and they are very comfortable with it and they are doing it. But previously before the midwives were taught, we’d pile these mothers on the ward until the doctor would come (…) And most of the abortions would end up being septic even when they wouldn’t have to (…) But when we sense dangers of complications that’s when doctors come in to assist with the problem.* (Head nurse/midwife, 15 years’ experience)

Doctors had divergent perceptions of the responsibilities in PAC. An understanding that midwives only performed MVA under the supervision of, or in consultation with, a doctor was prominent among some doctors, and this was perceived as good practice. On the other hand, other doctors were aware of and encouraged midwives in the autonomous provision of PAC, including MVA. A doctor ought only to be consulted when necessary.

*…Previously it (evacuation) has been the doctor’s procedure but now so many midwives know how to do it (MVA), if the pregnancy is more than 12 weeks, we give them miso and she’ll have a spontaneous abortion.* (Midwife, 15 years’ experience)

The PAC was arranged differently in each health facility. Doctors sometimes perceived PAC as poorly organized or non-existing, explaining how they would find women with abortion complications among women in labour on the ward. Midwives explained that PAC was available, but not always in a specified location, so PAC patients could end up in different wards and be taken care of by different midwives, who were more or less qualified or specialized. PAC counselling was not always available, commonly through a lack of time and means for proper provision. However, providers understood the importance of counselling, and improvements in the means for counselling (training, methods and privacy) were requested.

*Nowadays me I don’t see it (PAC) anywhere, but there were some staff some time back who were trained, but they are no longer working here…hmm, transferred to other Health centres.* (Midwife, 23 years’ experience)

There was generally a positive attitude towards task sharing. Some hospitals had shifted almost all responsibility to the midwives, whereas others shared tasks among the available staff. The doctors would provide services when they had time, but all informants stated that doctors were rarely available, and that this resulted in delayed service provision while waiting for them. There was a general perception of increased efficiency in service provision when tasks were shared. Midwives felt that the quality of care was equally good or even better when provided by them rather than doctors, since the doctors would commonly be out of practice and brought in from outside, with no context. Doctors agreed that the quality was equal when the care was provided by midwives, if they were properly trained and supervised, but opinions such as that the service was even better when provided by the doctors themselves were communicated. Moreover, informants agreed that midwives, if trained, should be enabled to provide misoprostol as well as MVA. Nevertheless, some barriers to misoprostol provision by midwives were clearly seen among the doctors, and one doctor highlighted that midwives themselves feared providing misoprostol due to restrictive policies and would not want to be held responsible if there were complications.

*But I could imagine, if I was to handle the patient myself, it would definitely be better than the midwife. Especially, since they use the MVA and I don’t know how many have specific training for that, I don’t know.* (Doctor (ob./gyn.), 14 years’ experience)

*Hmm, if they are trained well, they have been given the skill, there won’t be a difference. But if they are not, then there can be a difference because they may not have the real skills, hmm.* (Doctor, 15 years’ experience)

*Miso they (midwives) also give, they just fear but they give. (Interviewer: Why?) It’s a feared drug in the country. It’s not yet recommended. So you prescribe it at your own risk. So it’s only approved for prevention of postpartum haemorrhage, not for abortion, so you just prescribe it at your own risk. If you can defend it*. (Doctor/intern, 1 year’s experience)

When asked about the most difficult part of PAC, midwives were frustrated that they were not able to help all women due to a shortage of equipment, an absence of doctors and a lack of skills. This led to unnecessary maternal mortality and morbidity.

*When you are not skilled and there’s no doctor around, just a simple MVA to help this mother to stop bleeding (…) So you find our patients are at risk of losing their lives.* (Midwife, 18 years’ experience)

## Discussion

This study highlights the lack of skills and up-to-date guidelines for the provision of adequate quality PAC at district level in Central Uganda. Moreover, our findings identify midwives as the main providers of PAC. Providers identified barriers to provision of quality care as the lack of in-service training, poor supervision, and too heavy a workload to manage PAC in addition to other services. Moreover, our study identified a need for training in the use of MVA and misoprostol for uterine evacuation and training in contraceptive counselling in the context of PAC.

Experience and training seems to be crucial factors determining the willingness to provide safe abortions. The providers expressed conflicting opinions when discussing legalization and provision of abortion. Providers with experience or in-service training, or both were the ones that preferred performing an abortion rather than rejecting a woman in need. Those with a negative attitude towards abortion would describe it as sinful and as nothing they could support. These providers were more likely not to have much experience or training. This is in line with findings from an Ethiopian study where providers with experience of safe abortion were 2.5 times more likely to have a positive attitude towards abortion than those without any experience [29]. Several studies highlight the importance of education and in-service training to improve attitudes and furthermore the quality of abortion care [25-28]. Similar to our findings, a study conducted in Vietnam sheds light on the contradiction between cultural norms and the reality facing health care providers in the context of adolescent SRH [30]. It is recommended that education and training addresses this controversy specifically [42].

Frustration provoked among providers in the provision of PAC was mainly explained by the lack of skills and resources. A major concern among informants was the limited training in the use of safe methods for uterine evacuation and contraceptive counselling in a post-abortion context. Sources of frustration among the midwives were mainly derived from their lack of skills and knowledge of how to care for women with incomplete abortions, in the absence of a doctor. Other barriers to quality PAC, as identified by the informants, were the lack of space and privacy, and the shortage of supplies, equipment and contraceptives. Apart from the issue of the lack of human resources pervading the majority of health services in low-resource settings [21], similar barriers to PAC have been identified in other studies. Barriers such as sustainable access to MVA instruments [43], as well as poor contraceptive counselling and lack of available contraceptives [44-46]. Moreover, barriers to misoprostol use in PAC refer to restrictive drug policies as well as poor access and affordability of the drug [19,20,22,47]. Misoprostol has recently (2013) been approved for post-partum haemorrhage and incomplete abortion in Uganda. It is therefore important to develop a strategy for effective implementation for the use of misoprostol in PAC. The use of misoprostol for uterine evacuation is believed to result in more accessible and improved quality PAC [8,10].

Our study highlights midwives as the main providers of PAC in terms of assessment, care and uterine evacuation in the first trimester. Midwives reported to mainly use MVA for uterine evacuations, however they also mentioned use of D&C and misoprostol. The rare use of misoprostol in PAC at district level was explained by providers’ limited skills in the medical treatment of incomplete abortion. Additionally, lack of stock and absence of hospital guidelines in the use of misoprostol were mentioned. Similar factors have previously been identified as limiting the provision of other SRH services in Uganda [33]. Interestingly, the national medical store in Uganda has reported an overstocking of misoprostol since 2011, and non-existent demand from health facilities [48]. Misoprostol is effective for the treatment of incomplete abortions [8,18,49] and feasible in resource-limited settings where surgical treatment is largely unavailable [16,50]. In addition, a study from Uganda shows no difference in effectiveness between the use of misoprostol and MVA in the treatment of incomplete abortion [15]. In line with recommendations and evidence from previous studies, our study emphasizes the importance of specific in-service training to scale up the use of misoprostol to improve PAC in Uganda [18,22,24].

According to the WHO guidelines of PAC, contraceptive counselling ought to be included [5]. The providers in our study understood the advantages of providing family planning in PAC. However, they felt that they were unable to provide it due to the lack of human resources and in-service training. Further reasons that were given are the absence of hospital guidelines and a lack of structure (the facility set-up and resource allocation) to provide contraceptive counselling in the context of PAC. Studies show that accessible family planning can prevent repeated unintended pregnancies and abortions. Still, contraceptive counselling is the component of PAC that is commonly overlooked [44-46]. Integrating the provision of family planning methods into PAC has been suggested as a way to decrease the number of unintended pregnancies [51-54]. Hence, it is of great importance to support PAC providers in their provision of contraceptive counselling.

A key finding of our study was that midwives felt that PAC was a main contributor to stress in general. This was particularly in terms of additional workload and the difficulties involved in the consequences of unsafe abortions. Doctors however, did not seem to perceive PAC as major contributor to their workload. This emphasizes the extent of the midwives’ role in the provision of PAC. In terms of roles and responsibilities doctors and midwives had different perceptions. Doctors believed that midwives did not conduct MVA without supervision, whereas midwives reported that they did conduct MVA generally when doctors were not available. Doctors would appreciate when midwives took responsibility and perceived that efficiency would increase if tasks were to be shared. However, doctors emphasized the importance of training midwives prior to task sharing. Other studies from Africa show that task shifting contributes to more sustainable, cost-effective and accessible health care [23,24,55-58]. Furthermore, the provision of first-trimester abortion utilizing MVA or misoprostol has been technically feasible for mid-level providers for years. When policies and laws are liberal, task shifting can increase women’s access to safe services [24]. Due to the restrictive abortion laws in Uganda, doctors in hospital settings are the main providers trained to perform safe abortion. This also limits access to safe treatment after an incomplete abortion, especially in remote areas [39]. A study conducted in Uganda reports an informal amount of task shifting as a response to shortages of staff. This has resulted in dissatisfaction among mid-level providers due to the increased workload and the persisting limited resources [22]. The SRH policy (2006) in Uganda enables midwives and clinical officers to provide PAC [38]. This study suggests that task sharing occurs in the clinics, but not in an organized way with clear guidelines, training and infrastructure. Moreover, the present sharing of tasks is partial, since midwives seldom administer misoprostol themselves. This, unlike MVA, was perceived as the doctors’ responsibility.

This study is focusing on PAC, however its implications may be applied within other areas of health care in Uganda. Understanding the sources of frustration among providers and their perceptions of barriers to provide quality care are crucial factors that need to be considered in the development of new policies as well as the implementation of present policies. Improving PAC is an important intervention to decrease maternal morbidity and mortality in Uganda. It is also a step towards a more equitable health care with focus on women’s reproductive health and their own choices.

### Methodological considerations

The emergent nature of the study allowed each IDI to have its own individual character. Focus Group Discussions (FGD) were considered, however due to logistical barriers such as long distances between the facilities and little availability of PAC providers, FGDs would not have been feasible in the context. Interviews were conducted until no new data were encountered and saturation was reached. The data from each IDI was rich, explained by the good and safe environment created on each occasion. The interviews were carried out in English, the working language of health workers in Uganda but a second language for most people. It was therefore important to consider potential misunderstandings, even though no obvious problems were encountered. The fact that the interviewer was a young Swedish woman could also have influenced the responses, but this problem is thought to have been circumvented with the presence of the Ugandan research assistant. The role of the research assistant was to take notes and to help with any language difficulties. Her involvement appeared to create trust between the interviewees and the interview team and thus is reflected in the rich data. The interview guideline was pre-tested, revised and validated through pilot interviews and peer review. The credibility was further strengthened by quotes highlighting the findings. The data were continuously analysed by a group of individuals with different cultural and professional backgrounds, to strengthen dependability. Additionally, it is important to consider the researcher as an interpreter in terms of the construction of meaning in the research process. To ensure reflexivity, the transcripts were read several times, the data were discussed and supervisors were consulted. The study was conducted in governmental hospitals in the Central Region of Uganda, but transferability of findings to other regions of Uganda as well as context-similar settings is likely to be relevant.

## Conclusions

Task sharing in PAC needs to be scaled up in order to implement the existing policy in Uganda. In-service training of both doctors and midwives, including methods for treatment of incomplete abortion (MVA and misoprostol), contraceptive counselling in the context of PAC and value clarification are needed. Educational curricula for nurses and midwives need to be revised in order to promote task sharing in PAC, including contraceptive counselling. Strategic planning among policy makers in relation to effective implementation of task sharing and the scaling up of misoprostol use for uterine evacuation would improve the quality of care and accessibility of services. This would result in a reduced abortion-related mortality and morbidity.

## Abbreviations

D&C: Dilatation and curettage; FGD: Focus Group Discussion; HIV: Human immunodeficiency virus; IDI: In-depth interview; MMR: Maternal mortality ratio; MVA: Manual vacuum aspiration; Ob./Gyn.: Obstetrics/Gynaecology; PAC: Post-abortion care; SRH: Sexual and reproductive health; WHO: World Health Organization.

## Competing interests

There are no competing financial or non-financial interests involved in this study.

## Authors’ contributions

MP contributed to the conception and design of the study, applied for ethical approval, collected data and is the main interpreter of the data, drafted and approved the submitted manuscript. KGD revised the manuscript for intellectual data and approved the submitted manuscript. CK contributed to the conception and design of the study and its revision for ethical approval, the data collection and the interpretation and analysis of the data, and approved the submitted manuscript. RN contributed to data collection, peer review and interpretation of data, and approved the submitted manuscript. MKA contributed to the conception and design of the study, the data interpretation and analysis and the revision of the intellectual data, and approved the submitted manuscript. All authors read and approved the final manuscript.

## Authors’ information

MP conducted the study as a Master’s thesis in Global Health at Karolinska Institutet in Stockholm, Sweden. She has a background in biomedicine (BSc) and has previously worked with SRH in Mozambique and Nigeria. She is currently pursuing her PhD studies in abortion-related research in India. KGD is a professor and senior physician at the Department of Women’s and Children’s Health at Karolinska Institutet in Stockholm, Sweden, with previous experience in Uganda and several other African countries. CK is a medical doctor with an MSc in Obstetrics and Gynaecology at Mulago National Referral Hospital in Kampala, Uganda. RN has a BSc in Public Health from Makerere University, Kampala, Uganda. MKA is a midwife and a research scientist in the Department of Women’s and Children’s Health at Karolinska Institutet in Stockholm, Sweden, as well as a lecturer at Dalarna University in Falun, Sweden. She has previous experience in Uganda as well as in other African and Asian countries.

## Pre-publication history

The pre-publication history for this paper can be accessed here:

http://www.biomedcentral.com/1472-6963/14/28/prepub
